# Valedictory Address Delivered to the Graduates of the Baltimore College of Dental Surgery, at the Commencement, Held March 28th, 1851

**Published:** 1851-07

**Authors:** Eleazar Parmly


### ARTICLE IX.

*Valedictory Address Delivered to the Graduates of the Baltimore College of Dental Surgery, at the Commencement, held March 28th,* 1851. By Eleazar Parmly, M. D.

Gentlemen Graduates :—It is a matter of no less regret to me, than it is to you, that illness has prevented Dr. Harwood, of Boston, from discharging the duty assigned to him for this occasion. We were all rejoiced that the appointment was so cheerfully accepted at the time, and delighted with his kind response when the request was presented to him. Another cause of no less regret to you, to the faculty and to myself, is the absence of Dr. Gardette, of Philadelphia, (occasioned by the severe illness of Mrs. Gardette,) who has for several years honored this institution with his presence, and cheered the hopes of the graduates by his kind and hearty approval of their efforts and creditable attainments in their professional studies. With the expression of his regret at not being able to be present, he desired me to express also his best wishes for all who are in any way connected with the institution.

Under these unexpected circumstances, it was not without hesitation, that I consented to speak to you, remembering how ably and with what benefit to the graduates, and to the profession, and with what thrilling interest and pleasure the audience listened to the discharge of that duty last year, in the address of that bright ornament of science and of the dental profession, Dr. Hullihen, of Wheeling.

I knew how much the faculty, the graduates and the profession had a right to expect, and would have received, from the able pen of Dr. Harwood, who, independently of his high attainments in all that appertains to medical and surgical science, has been one of the most highly distinguished men in practical dentistry known to our country—when I say *that*, it will be understood that he is not surpassed, if equalled, in any other country.

The deductions from such knowledge, and from such experience, would have been a rich feast to you, as well as to myself, and would have furnished a bright star from an eastern sky to be placed opposit the dazzling one we beheld last year from the west, to cast their radiance upon each other, thereby developing more perfectly the beauty and brilliancy of both.

It seems proper that the last advice given to you on leaving these halls of study, to enter upon the practice of your profession, should come from just such sources, and from just that kind of knowledge and experience which have enabled their possessors to overcome all difficulties, and place themselves at the very summit of professional distinction, and at the very head of social relations and intellectual associations; and I wish that I could cast one ray of light upon these polished columns of our professional edifice, to be reflected upon you, and impress your minds with the truth, worth, and consistency of their individual and respective characters.

Those of us who have passed through the difficulties of successfully establishing ourselves in professional life, can deeply and sincerely sympathise with you in the mingled feelings which must necessarily crowd upon you at this interesting and eventful period. The bright and joyous hopes of success—the dark and desponding fears of disappointment, each in their turn, gaining the ascendancy over the mature thought and deliberate calculation. The pain and regret at separating from your respected instructors, and fellow students, probably to meet no more on earth—your impatience to commence the arduous duties of your profession—the doubtful suspense, the formidable opposition, and the severe scrutiny to which your practice and your labors will be subjected, must be common to you all, and give rise to conflicting emotions in the bosom of each one of you, who are now about to engage in the conflict of professional life, which will either result in the attainment of honor and fame, bringing credit upon yourselves—pride and gratification to your friends—praise and distinction to your teachers, and honor to this college. Or, on the other hand, in that ignoble defeat which will involve not only your own character, but the feelings of your friends, the good will of the profession and the good name of our cherished institution. Let these considerations, my young friends, stimulate your exertions—excite your ambition, and lead you to prefer virtuous industry, with its slow but sure and just rewards, to the uncertain glitter which folly, fashion and extravagance may throw around you, and which may ultimately allure to hopeless ruin. Avoid then, I pray, as you would avoid disgrace, despair and death, every kind of licentiousness and unbecoming dissipation, for they often, in their effects, lead to results, which to friends, family and reputation, are as terrible and as much to be dreaded as death itself, in its worst consequences. There are very few if any professions in human society, in which sobriety, purity, honesty, industry and untarnished honor, are more essential to professional success than that of dental surgery.

You have now completed your course of preparatory study and your examining committee have, with pride and pleasure, witnessed the creditable manner in which you have sustained yourselves under a careful and rigid scrutiny—you have had conferred upon you, by the authority of the state and of this college, the degree, which, by legislative statute, declares you "Doctors of Dental Surgery." The very title implies that you are thoroughly acquainted and carefully instructed in the sciences which more immediately have a bearing upon, or are connected with your future practice as dental surgeons. And it will become you so to demean yourselves in the discharge of all your duties as to convince the world that the title has not been unworthily bestowed. This diploma, which is an evidence of your qualifications, will serve you as a letter of unlimited credit, to draw at will upon the confidence and support of your fellow citizens; and may no act of yours cause this credential to be slighted or dishonored, or those who have confidently subscribed their names to it in your behalf, to be made to feel that unworthiness attaches to any one bearing the honors of this college, which, rightly regarded and improved, will be a passport to future success, and a perpetual recommendation to you throughout the world. But its influence cannot be maintained without, on your part, a strict correspondence of life and conduct, and that correspondence will consist of, and depend upon, your professional intelligence—your energy, application, and the strictest purity and integrity, and upon your careful investigations into what is true and what is false in practice—discarding the one and pursuing the other in despite of all opposition, and all prospect of gain, and let every day's practice add to your stock of knowledge.

It is but a few days since I heard a lecture in New York, from the justly celebrated Mr. Silliman, professor at Yale College, who informed his hearers that he had witnessed the rise and progress of the science of geology in this country, from its earliest dawn to the present day, and that his own life embraced the whole period of the existence of that science as a branch of collegiate study. Many of us can say the same in relation to dental surgery as a science, for it was not until the establishment of this school that any organized system of study was observed, or even thought necessary by the public ; yet there is no department of learning in which greater advancement has been made, than in that which relates to dental practice, and I cannot better illustrate this truth than by saying, that after I returned from Europe to New York, probably, in the year 1822, a lady, in speaking the praises of her dentist, one, too, who had ranked the first in that city, said, that he had never studied the profession, nor for him was it necessary, as he was a "*natural dentist.*"

This will give you an idea of the existing state of dental science, and of public opinion in relation to it in New York, when it was at that time commonly supposed that "pulling teeth" was the chief employment of the dentist. I am sorry to say, that these "natural productions" have not altogether ceased growing, either in the dental or in the medical profession. Such is the fortunate condition of our highly favored country, that a rapid growth or increase is going on ; and such is the accommodating disposition of some of our kind hearted people, that they swallow every thing that comes in their way, and permit their dentists, with or without recommendation, to fill their teeth with all sorts of mineral substances, from crude mercury down to pulverized stone—and their doctors to fill their stomachs with all vegetable mixtures, from the balsam of the beautiful and majestic wild cherry, down to the almost inconcievably small seed of the modest and shade-loving lobelia, and thus rejuvenate themselves, in thought, at least, on "life pills" and "phenix bitters." In our own department, this natural state of things has very much improved since the introduction of amalgam, whose remarkable transforming power has been known to change, in a short time, such men as rank with horse jockies, watch thieves and bigamists, into good and honest, as they are called, "Amalgam Dentists."

That natural abilities, unaided by the advantages which you have had, with close observation and long experience in a series of years, has produced some of the ablest men, both in the medical and dental profession, I will not deny, and they have also furnished science with some of its brightest ornaments. But I do not believe in the efficiency of those "natural dentists," "natural bone setters" and "natural doctors," in the beginning of their *unnatural* operations. That knowledge which will generally lead to success, must be grounded in an intimate acquaintance with the anatomical, physiological and mechanical structure of the organs upon which we are to operate. The rapid advance which has been made in medical and surgical science has depended upon this knowledge, and great have been its achievements and glorious its triumphs. It is that knowledge which enables the skilful surgeon to restore the distorted limb to its fair proportions ; to banish, with a touch from his practiced hand, obliquity of sight, and place the organ of light, of beauty and of vision in its right position. And it was that knowledge which enabled our distinguished cotemporary, Dr. Hullihen, to reduce a face marred by hideous deformity, to comparative beauty, symmetry and comeliness. And it is that knowledge that will enable you, even in the beginning of your practice, to avoid the injuries inflicted by ignorance and presumption, and confer the benefits which the combined knowledge and experience of your predecessors have found most successful, and which has gained for them the gratitude of thousands, and placed the profession on the high ground it occupies in this country over any other.

A distinguished Parisian, a gentleman and scholar, Count de D., now in New York, remarked to me a few days ago, that he had "discovered, during his residence in this country, that there are here two clases of dentists—one very high, and the other very low; and that the latter lives upon the reputation of the former." This latter class consists chiefly, at the present time, of amalgamists. There may be honest and truthful men among them, but if there are, they differ very much from those champions of amalgam whom I have encountered; for I have already proved some of them to be without either professional or moral honesty, and can do the same in regard to others, if necessity shall require it. I am willing, however, that this necessity shall never call me to this unpleasant task; but I will never shrink from the task, unpleasant as it is, when the conduct of knaves and charlatans shall render that task a duty. To the American public I owe many obligations for the confidence my fellow-citizens have reposed in my professional practice, and I intend to discharge, at least, a part of these obligations, by exposing the tricks of mountebanks and the impudence of knaves. Of all the quackeries of our profession, or of those who live only to disgrace it, I regard mercurial paste as the most notorious, execrable and base, as it has been used in this country; and should a part of my life be spent in exposing the evils of such nostrums, I shall not regard it as utterly lost to my race, or to my country.

In our intercourse with the world, we meet with two distinct classes of men. On the one hand, are those who earn their livelihood by industry and labor; and on the other hand, are those who live by the industry and labor of others. So, in the former of these classes, there is a distinction not less strongly marked in those who exercise callings which are useful to mankind, and those whose avocations are either unimportant or detrimental to society.

Without entering upon any details upon this subject, which might be deemed partial, it is not improper that on an occasion like the present, we should congratulate ourselves on belonging to a profession which is not only eminently useful, but highly important to the interests and well-being of mankind; although we are, in some degree, forced to admit, that in many instances, its actual importance results especially from the follies and vices that exist in the excesses of what is called an advanced and refined state of civilization, over which morality and purity have neither secured nor diffused the blessings of health and virtue. But whatever be the cause of these evils, it is, nevertheless, one unfailing cause of the gratification which our professional labors bring with them, that they mitigate the actual suffering and augment the positive pleasures of human life. This, as we have said, is by no means true of many of the ordinary avocations, in the exercise of which numbers of our citizens acquire fame and fortune. That we labor, then, in a useful calling, in the service of our fellow-men, which is the truest service assigned us by heaven, should be a never-failing source of individual satisfaction and social congratulation in our profession. If it were not an eminently useful profession, the duties of which, when properly performed, are works of charity and benevolence, I am persuaded, there are many individuals who now labor in its ranks, who would abandon it forever; and this is, probably, the true reason why dental practitioners, as well as practitioners in other branches of the healing art, continue the labors of their profession longer than their personal convenience, or the inducement of pecuniary emolument would otherwise impel; and yet it is not to be doubted that both personal satisfaction, and the recompense of reward are entirely proper elements to enter into every man's attachment to his profession. Men live together, and our social relations are mainly based upon mutual usefulness, protection and support—and mutual usefulness, implying reciprocal offices of kindness, is but another name for mutual compensation.

Thus, a professional man has daily the opportunity of showing whether he has respect to the highest attainable or commanded virtue, that of loving his neighbor as himself; and by a faithful and honest discharge of his duties, he has also the daily and hourly opportunity of fulfilling the divine precept, of doing unto others as he would that they should do unto him.

We have indulged in the foregoing remarks, merely to keep before the mind the all-important fact, especially in the opinion of every honest man, that whilst we secure to ourselves a livelihood by the daily labors of our calling, we do it without violating our sense of justice or our morality, by exercising a profession which merely tends to the corruption of society, or, at least, supplies only its imaginary or unreasonable wants. This fact, alone, is quite sufficient to place us at ease with our profession, provided it compensates our efforts with a suitable remuneration and support; and we, on our part, return to society a just equivalent in faithful and skilful operations.

These reflections apply not more aptly to any members of the dental profession as such, than they do to many of the graduates and students of this college, an institution which, in realising our fondest expectations, has greatly gratified our professional self-respect. It is gratifying to us that the cause in which this institution is engaged, is unequivocally and undeniably the cause of humanity; and that all the knowledge and skill which have been here gained and have issued from it, are among the works of philanthropy and good will, being no other than fraternal acts embodied and imparted in the love of truth—in the desire to advance the interests of the profession, by the brotherhood of kindred minds and kindred sciences.

With firm footing, therefore, upon this good ground, we have only to go forward, step by step, with the onward march of the age, and place our favorite institution and all its abettors among the foremost benefactions of our race—as certainly as we do this, we shall secure to our country perpetual improvement in professional science and dexterity. Of this term, "dexterity," I would emphatically say a few words to the students now before me. It is a word I wish you particularly to understand, in our vocabulary, signifies skilfulness of the right hand; it is a term, therefore, peculiarly expressive of one of the indispensable characteristics of a good dentist. Without science, it is very easy for a practical dentist to do much harm; and without manual skill and dexterity, it is impossible for him to do much good. This is a point I have always urged upon the consideration of the profession—and the longer I am in practice, the greater need do I see of enforcing and insisting upon its claims, from the awkward and unfinished manner in which some of our prominent men, claiming to be "medically educated," advocates of cement, gutta percha and amalgam, leave their operations. Nor does this circumstance any more degrade our science, as considered by some, into a mere manual art, than the fact, that a good musician must have the required skill in fingering the keys or strings of his instrument, as well as a thorough knowledge of the science of music, in order to become, or to be rated, a good performer or perfect musician. Both "theory" and "practice," therefore, are indispensable (and, perhaps, equally so) to the success and reputation of our profession—and those are both taught with equal ability and care in this collegiate institution—a fact which has never yet been equally true of any course or system of private tuition, for which the studies and manual exercises of this seminary are intended as a substitute.

As the hardest and most valuable productions of nature and art are slow of growth and perfectibility, but of great longevity and duration, so must we expect it to be with this institution. Should it survive its infancy and pass from the hands of its present teachers and professors to the care of others and posterity, it will remain as it now is, the first as well as the oldest institution of the kind in the habitable world, as none have been known to precede it since the dawn of human history—and but one other, as far as I am acquainted, now exists, which has been established on the same basis for the benefit of the great West, by the energy and enterprise of our brethren and friends in Cincinnati, and which we hope will do much that will be of incalculable benefit to those who are desirous in that section of our country, of qualifying themselves for general practice and usefulness in the dental art.

This college is acknowledged by, and holds a representation in the largest and most respectable body of professional men that this or any other country ever assembled together, and the cordiality and kindness with which the delegation of this institution was received as such, is, and should be, a cause of pride to ourselves, as well as of grateful acknowledgment to the high-minded and generous philanthropy of those who gave that reception. The voice of admiration and approval of its great advantages and happy influences reach us continually from other nations beyond the seas. Prospects like these cannot fail to encourage us to unwearied effort, and from the success which has already attended practitioners, both in the new and old world, capable of giving dignity to any human calling, with colleges, societies, essays, periodicals and journals, and finally, Dentalogia, to celebrate the triumphs of their skill to generations yet unborn. Such is the condition of the profession, young gentlemen, to which you have attached yourselves, and among whose members now become fairly and honorably enrolled, and may you do justice to yourselves, to the institution from which you now go, and to your teachers, as a return for that good which you so generously and frankly acknowledge they have done to you. Knowing the great advantages you have here received, it is a duty which you owe to the institution to increase the number of its graduates by earnest recommendation, by which you will add honor and dignity to the profession, and confer a lasting benefit on mankind. This is no more than gratitude and philanthropy demand at your hands, and no less than a common sense of justice will impel you to perform.

Since we last met, two members of the dental profession well known in this country, have been removed by death—and it becomes us on occasions like the present, kindly and respectfully to remember those who have given character to the profession, either by their moral or professional standing—by their influence in social relations on society—or who, by their talents or writings, have contributed in any way to our stock of professional knowledge. We should all aim to leave something behind us worthy of being remembered, and worthy of being told to our successors.

Leonard Kœcker was born the son of a Lutheran clergyman, in the year 1785, in Bremen, in the kingdom of Hanover. In early life, he received the rudiments of a good plain education, but nothing more. At the age of fifteen he was engaged in a mercantile house in his native place, and during his stay there, he became acquainted with a peddling Jew dentist, who took much notice of him, and allowed him to become familiar with his instruments—and who also gave him a small set of implements which are now in the possession of his son, a young gentleman of fair professional promise, who has chosen his father's calling, and is settled in Philadelphia. It is to him that I am indebted for these facts, communicated to me in a conversation a few days ago. His father's childhood was marked, as stated by a lady who knew him as a child, by great gentleness of manner and was even then uniformly polite and kind; all which characteristics continued through life, and to that suavity of manner much of his success may be attributed. He came to this country as a commercial agent, and landed first in this city. The agency soon failed, and he was left with very limited resources, but being an accomplished musician, and mixing much in society he made many friends. On visiting Philadelphia he made his circumstances known to Mr. Delaplaine, representing to him the knowledge he had obtained from his friend the Jew, and was advised by that gentleman, immediately to commence practice with the knowledge he then possessed, and to add to it as opportunity might offer. He accordingly took a room in Sansom street, and his first visiter was a gentleman who called to have a tooth extracted. He grasped the tooth with an instrument, shut his eyes and turning his head from the patient, made a strong effort, which dislodged the tooth from the socket, but under such excitement that he knew not whether the tooth was out or the jaw broken. The gentleman complimented him by saying he had never before had a tooth extracted so easily, and from that gentleman's influence his success commenced in Philadelphia. At that time there were no dentist instrument makers that he could employ, and he made his own instruments as necessity suggested the want of them, which practice he afterwards continued as far as supplying patterns. At that time gold foil was but imperfectly prepared, which he used to anneal himself. His business continued to increase in Philadelphia until it reached $8,000 a year, but from dyspepsia his health failed, and he was obliged to give it up, and went to Europe in 1822. Although he took with him the best professional recommendations, and socially the best introductions, he had many difficulties to contend with in establishing himself in London—but by unremitting attention and by gaining the favor of distinguished professional men, he finally succeeded in obtaining a steady practice, worth as much yearly as it was in Philadelphia. By application and study, he became a good Latin, French and English scholar, and received diplomas from the medical colleges of Philadelphia and London. After a residence of five years in London, he produced his "Principles and Practice of Dental Surgery," which was severely handled by the London critics, notwithstanding it was the best and most complete system of dental practice that had been given to the profession.

About this time he effected a perfect cure of a diseased palate, and supplied, successfully, its loss, for a gentleman who had been recommended to him by Mr. Laurence and sir Astley Cooper, the great English surgeons, which circumstance secured to him the confidence, friendship and respect of those gentlemen. He had an ardent love for his profession, and after practicing about seven years in Philadelphia and twenty-eight in London, he retired, selling the good will of his practice for twenty-five hundred pounds, about twelve thousand dollars. He was a man of refined taste, a lover of the fine arts, and especially of music, but of very extravagant and expensive habits. Speculative in his business transactions, he, at one time, lost nearly fifty thousand dollars, the best part of his savings, by unfortunate investments in rail road stock. About nine months before he died he met with a serious accident, from his night gown taking fire, by which he was dreadfully burned, and from which he never recovered. He continued to fail from this injury until dropsy and asthma supervened, which terminated his life at the age of sixty-five. He was buried on the 8th of August in Norwood Cemetery, about six miles from London.

Of the parentage and childhood of Dr. Lewis Roper, I have not been able to collect any important facts, farther than that he was born in Philadelphia; he was the son of a sea captain, who was lost at sea when he was a child. At eight or nine years of age, he was placed with Mr. John Sillers, of Upper Derby, a farmer, with whom he lived until he was apprenticed to Mr. Ferris Price, a house carpenter in Philadelphia, where he stayed until he was twenty-one, at which time he commenced business for himself. Before, however, he had finished his apprenticeship, he married the daughter of Mr. Jonathan Shoemaker, which marriage was in one year terminated by the death of his wife. Being now without the responsibility of a family, and free to follow the bent of his inclinations, he left Philadelphia and studied his profession in New York, with a dentist then residing there, by the name of Chevalier. After obtaining all the knowledge he could from him, he went south and practiced some four or five years, returning to Philadelphia in the year 1829 or 1830. He soon gained a position and acquired a considerable reputation as a dentist, and at the death of Dr. Hudson, in 1833, he took the dwelling previously occupied by that worthy man and highly distinguished dentist. In the mean time he received the diploma of the University of Pennsylvania, the first and oldest medical college in Philadelphia.

Dr. Roper told me, some years ago, that his first success in practice was accidental, and arose from his acquaintance with a family with whom he was fellow passenger on his first leaving his preceptor in New York. Learning that he was a dentist, they requested him to go to the place where they resided, Charlottesville, Virginia, and being a family of influence, their recommendation at once gave him a fair beginning in professional life, and he gained strength as he went from place to place. As it has been already said, he returned to Philadelphia, and with the avails of his professional success he was enabled to procure a good house, which he furnished very handsomely, showing much taste in its internal arrangement; he shortly afterwards married, in 1831, Miss Glover, the daughter of captain William Glover, of Philadelphia, a young lady who had been carefully educated, of refined intellect and of great personal worth and domestic virtues, a marriage of the utmost cordiality and connubial happiness.

After a few years of great prosperity, Dr. Roper, in 1840, was called to mourn the death of this excellent woman, which occurred almost without warning, from the rupture of some important artery or blood vessel, leaving one only daughter.

Dr. Roper had by this time obtained a large practice and saved a handsome sum of money, but was encouraged to place it in the hands of some of his friends, who were dealers in stocks, for which they made him large purchases. At one time, the stocks increased upon his hands which added greatly to the sum already invested. This encouraged him to go on until a large portion of his hard earnings were by these stock operations completely swept away. Dr. Roper, in 1842, married a third time, a lady of this city, a daughter of Thomas Hillen, Esq. one of the most respectable citizens of Baltimore, a lady of great personal worth and attraction.

He purchased a splendid house in Philadelphia, furnished it with great taste and elegance, and lived in a style corresponding with the establishment. Dr. Roper had a great love for paintings and works of art. His collection would compare well with the collections of other gentlemen of acknowledged taste and liberality in that way. He had the largest collection of valuable and rare coins and medals, that I have ever seen in any private cabinet, and I should think, that his collection of autographs would compare with the largest in the country, all of which were arranged with great care and labor. In the midst of all these quiet and social comforts and advantages, the California excitement broke out, and with his enthusiastic spirit and ardent temperament, he entered largely into the purchasing of property, consisting of houses and household conveniences, for that market. After shipping his merchandise in Philadelphia, he took passage at New York in September, 1849, in one of our fine steamers, full of hope and expectation, to be realized in the newly discovered world of gold. On his arrival there he made purchases of ground, sold some of his houses and erected others, and at one time seemed to have made a large fortune, but a change came over the city of San Francisco, by fires and other causes, and a vessel containing seven houses of his not arriving, Dr. Roper lost the whole of his large investments, and with broken hopes and humbled ambition, took passage on the steamer Panama, for his native land, and died of cholera on the very day the steamer arrived at Panama.

A fellow passenger, lieutenant Hoskins, who attended him most faithfully, says, that after the cholera broke out, Dr. Roper was continually with the sick and dying, and that he really fell a victim to his devotion to their necessities.

On the morning he was taken, 21st August, 1850, he had been administering to their wants, and being much exhausted, he went to the upper deck about ten o'clock, and laid down to rest upon a settee, where he was immediately seized, and so sudden and so violent was the attack, that he soon became insensible, and at seven o'clock the same afternoon died. As soon as preparations could be made, the passengers assembled on the side of the vessel's deck, and performed the last sad duty to the lifeless body, by lowering it gently into the water with no less coffin and winding sheet than the boundless billow, and no less grave than the vast and almost shoreless Pacific, whose rolling waves murmured the mournful requiem as the remains of Lewis Roper sunk to their final resting place.

Dr. Roper was known through life as being a highly honorable, sensible, quiet and amiable man; he took great interest in whatever tended to advance the science of dental surgery, and his exemplary and moral life gave character to his professional standing. He was one of the original formers of the American Society of Dental Surgeons, and one of its first vice-presidents; he took great pains to be present at its meetings, where he was much respected; he was a member of the first examining committee, at which I was present, appointed by this college; his professional views and opinions were always treated with deference and respect, being always marked with thought, discrimination and judgment.

The biographies of these two individuals, and their incidents of life, will furnish you, my young friends, with valuable lessons which I hope will make a deep impression upon your minds—for in these two biographies, there is much to adopt and much to avoid. The industry, application, perseverance and gentlemanlike deportment of the departed will be examples for you to follow, and it is not certain that any one of you will surpass, if you ever attain in that respect the standard of either of them. In their business character the greatest fault, perhaps, of both, was a speculative disposition, which gave them a desire to accumulate faster than the steady avails of professional labor or lawful interest on stocks and loans would enable them to do. You would be safer in placing yourselves with careless operators, having dull heads, rough hands and sharp instruments, than to risk your money with reckless speculators, having oily speech, smooth hands and keen heads. The first can only expose you to a simple *break* or *cut*, which your knowledge and skill can soon cure or restore, but the other will *break* up your comforts and *cut* you out of all you possess, which no art or skill of yours can ever regain. I inquired of a gentleman who had made a large sum of money by steady business in a northern town, and who lost it in Wall street, how long a man of fortune, unacquainted with the business of that street, could sustain himself there? he replied with much emphasis, "if he is a very shrewd fellow he may possibly hold out two weeks—it took but a little longer than that to ruin me." Know, then, my young friends, that stock and all other speculative operations, are the very last things that dentists should meddle with, for like those whose experience is now before us, whose names we thus respectfully and kindly remember, you, too, will be likely, from such operations, to *extract* nothing but sorrow and disappointment, your coffers will be thoroughly *clean* of all *adventitious* and *local* deposits. Your accumulations from principal and interest, will be widely *separated* and effectually *stopped*. Your *plate*, whether in *single* or *double sets*, will no longer be *adapted* to your own use, but with a *painless grasp* fall into other hands; your very *substance* will be *wasted* and your credit *shattered*. Losing, thus, your professional *caste*, you will utterly fail to *regulate* the *derangement* of your affairs, occasioned from various *processes* having *absorbed* your interests by *local* or *established agents*, thus *breaking* up the *elastic springs* of youthful ardor and ambition; old age will come upon you, making the *double impression* of regret and disappointment at finding your pockets *poorly filled* with *base metal*, when they should be full, even *rounded* to their *edges* with *pure* and *polished gold.*

You are now about to take leave of a place that will, in after life, be hallowed with many pleasing thoughts, recollections and associations. The anxieties you have felt, the difficulties you have overcome, the friendships you have formed, the pleasures you have enjoyed—the very city itself, with its hills and valleys, its monuments and squares, will be subjects upon which your memory will dwell with unmixed pleasure.

Baltimore, so full of interest, kindly feeling and hospitality, is not an unmeaning picture with which to compare your professional life, for in its local patrons and advantages it is not unlike what your intercourse will be with mankind. Your experience will be to you, as ours has been to us—uneven, irregular, broken and diversified. Your professional practice will make you acquainted and familiar with high situations, generous aims, noble efforts, elevated walks, refined elegance, polished exteriors, great liberality—Amidst these bright traits of character, which apply with no less truth to the city than to the people, you will sometimes meet, and I hope but rarely, with *neglected ruins*, *round turns*, *sharp points*, *narrow ways*, *low grades*, *fancy railings*, *deep* and *cross cuts*. But, however many of these you may encounter, you have only to maintain steadily your position, keeping the pillar of fame constantly in view, which will be to you what the proud monument to Washington is to the citizens of Baltimore; and even amidst difficulties, elevate your thoughts to the summit of professional excellence. And there let them rest with the same feeling of pride that they indulge in gazing on the noble work, and resting their eyes upon the majestic form, on its summit, representing to them the perfection of human character, unsullied by the storm and unhurt by the tempest. It should, as it no doubt will be, a source of pride to you to say that you studied your profession in Baltimore. Baltimore, therefore, has a claim upon you—she demands that, bearing upon your credentials her proud name, you consider her glory identical with your own. Never, never can I forget the feeling of pleasure that thrilled through my whole frame, when from that monument's lofty height I first looked upon this beautiful city, and leaning against that noble structure on its top, I could not resist the desire to express, though feebly and faintly, the feeling that animated my thoughts, and should the reading of the expression of them excite no other emotion in your minds, it will at least be associated there with the day and with the occasion when you received your first professional honor, and with that association, I desire to be kindly cherished in your memories.

Washington! immortal name,
  On thy monument I stand,
Noble tribute to thy fame,
  Founded by a grateful land!
Yet—that land itself shall be
Noblest monument to thee!

Land by matchless valor bought,
  Home of nations—happy—free;
All her children shall be taught
  To respect and honor thee;—
Be her banner wide unfurled
Bearing freedom to the world!

Far from this majestic scene,
  Bursting fair upon my sight,
Where the northern mountains green,
  Glitter in the morning light;
Fast beside a murmuring stream,
Came to me life's infant dream.

Still, 'tis all my native land,
  Wheresoe'er I chance to be;
And I grasp with friendly hand,
  All who love its liberty.

Liberty by heroes won,
Led by thee, our Washington.

Who upon this marble tower,
 Reared the patriot's noble form;
Fraught with mercy's gentle power,
 'Mid the raging battle storm?
Your's the praise from shore to shore
Noble sons of Baltimore.

